# Leverage of quick-soluble gelatin microparticles in transarterial chemoembolization of canine urothelial carcinomas

**DOI:** 10.1080/01652176.2026.2679557

**Published:** 2026-05-29

**Authors:** Sunghoon Jeon, Gahyun Lee, Jin-Kyung Kim, Namsoon Lee, Dongwoo Chang

**Affiliations:** a Haemaru Referral Animal Hospital, Seongnam, Republic of Korea; b College of Veterinary Medicine, Chungbuk National University, Cheongju, Republic of Korea

**Keywords:** Interventional radiology, lower urinary tract tumor, quick-soluble gelatin microparticle, transarterial chemoembolization, urothelial carcinoma

## Abstract

Lower urinary tract carcinomas in dogs are rare conditions with a poor prognosis. This prospective clinical study evaluated the safety, feasibility, and therapeutic efficacy of transarterial chemoembolization (TACE) using quick-soluble gelatin microparticles (QS-GMP) in 29 client-owned dogs with lower urinary tract carcinomas. Tumors primarily involved the bladder and urethra. Following selective catheterization of tumor-feeding vessels, intra-arterial carboplatin was administered prior to QS-GMP embolization. All patients underwent follow-up computed tomography (CT) scanning 4 weeks post-TACE. Tumor volume decreased in all cases, with a median reduction of 76.41% (95% confidence interval: 65.06–78.41%, *p* < 0.001). Partial remission was observed in 96.5% of patients; one case showed stable disease. Clinical signs, including pollakiuria, hematuria and stranguria, improved. No major complications occurred during the procedure or within two weeks after the procedure, and minor adverse effects were self-limiting. Repeat TACE was performed in 28/29 patients. The median survival time was 569 days for all patients and 534 days when non-tumor-related deaths were excluded. These findings suggest that TACE with QS-GMP may be an option for dogs with unresectable lower urinary tract carcinomas, with potential for repeated interventions and prolonged survival.

## 1. Introduction

Canine lower urinary tract tumors, especially bladder or urethral (urothelial carcinoma) malignancies, are rare diseases with poor prognoses (Norris et al. 1992; Mutsaers et al. 2003; Axiak and Bigio 2012). They often present with hematuria, urinary incontinence, dysuria, and nonspecific clinical signs such as anorexia and weight loss (Vail et al. 2007). Tumor-induced urethral obstruction can result in urine retention (Axiak and Bigio 2012). Conventional treatment includes surgery, non-steroidal anti-inflammatory drugs (NSAIDs), chemotherapy, radiation therapy, or combination therapy (Fulkerson and Knapp 2019). Despite improving clinical signs and extending median survival time (MST), surgical intervention is often limited owing to its invasiveness, complexity, and high morbidity, especially for bladder trigone or ureteral tumors (Marvel et al. 2017; Bennett et al. 2018). NSAIDs and systemic chemotherapy are the primary medical treatments for canine urothelial carcinomas. However, maintaining a stable disease (SD) state rather than achieving rapid clinical improvement is the aim of these therapies (Knapp et al. 1994; Mutsaers et al. 2003; Fulkerson and Knapp 2015). Intra-arterial chemotherapy is a promising alternative to conventional intravenous chemotherapy in dogs with lower urinary tract tumors, including bladder carcinoma (Culp et al. 2015). Despite its superior tumor response rates, this approach requires repeated vascular interventions every 4 weeks, and data regarding its long-term outcomes remain limited.

Transarterial embolization (TAE) is used for the management of lower urinary tract malignancies in humans (De Berardinis et al. 2005; Loffroy et al. 2014; Chen et al. 2021). However, surgical resection remains the primary treatment option for lower urinary tract tumors, with TAE being used for managing intractable hematuria rather than tumor control (Chen et al. 2021). Conversely, in dogs and cats, TAE and transarterial chemoembolization (TACE) has been primarily limited to prostatic malignancies, showing excellent short-term tumor response and improvement in clinical signs (Culp et al. 2021; Pellerin et al. 2021). Nevertheless, the use of TAE or TACE for managing bladder and urethral tumors in dogs and cats remains limited owing to the risk of non-selective embolization, leading to bladder wall necrosis. TAE and TACE may serve as a valuable palliative therapeutic option in veterinary oncology, particularly for lower urinary tract tumors, provided ischemic complications are mitigated.

Recent human studies have focused on the use of quick-soluble embolic agents, which are associated with minimal adverse effects (Kawai et al. 2013; Sato et al. 2021; Alali et al. 2024). Quick-soluble embolic agents can effectively target and destroy tumor- or inflammation-induced neovascularization, achieving efficacy comparable with that of permanent agents (Pieper et al. 2015; van Zadelhoff et al. 2025). A recent preclinical embolization model study revealed that urinary bladder embolization with a quick-soluble embolic agent successfully induced vascular occlusion without causing serious complications such as bladder rupture (Jeon et al. 2025). Based on these findings, the present prospective clinical study investigated the feasibility, safety, and clinical outcomes of TACE with quick-soluble gelatine microparticle (QS-GMP) in dogs with lower urinary tract tumors.

## 2. Materials and methods

### 2.1. Case selection

All dogs included in this study were client-owned animals living under normal home conditions. Dogs diagnosed with lower urinary tract carcinoma at the Haemaru Referral Animal Hospital between February 2023 and November 2024 were prospectively enrolled in this study. After we obtained informed consent for treatment and data use for research, we treated all patients with TACE. The study protocol was approved by the Institutional Animal Care and Use Committee (2024003-HMR).

Inclusion criteria were as follows: (a) cytologic or histopathologic confirmation of lower urinary tract carcinomas obtained via traumatic urine cytology or cystoscopic biopsy, (b) availability of pre-procedural computed tomography (CT) angiography scans performed within 30 days before the initial embolization, and (c) no history of radiotherapy or surgical excision for the current tumor. Exclusion criteria included the presence of recurrent disease and administration of chemotherapy within 1 month before enrollment. Concomitant medications, including NSAIDs, antimicrobials, gastrointestinal protectants, anti-emetics, antidiarrheals, and dietary supplements, were allowed.

All enrolled patients underwent standard staging procedures, including physical examination, thoracic radiography, thoracic CT scanning, abdominal CT angiography, abdominal ultrasonography, complete blood cell count (CBC), serum biochemistry, and urinalysis. The initial TACE was performed within 30 days of clinical staging.

### 2.2. Case population

The 29 patients included 9 Malteses, 5 Pomeranians, 3 Toy or Miniature Poodles, 4 Chihuahuas, 2 mixed-breed dogs, 1 Yorkshire Terrier, 1 Shih Tzu, 1 Miniature Pinscher, 1 Dachshund, 1 Cocker Spaniel, and 1 Bichon Frise. The median age was 10.75 years (range: 5.67–15.42 years), and the median body weight was 5.03 kg (range: 2.6–13.0 kg). Of the 29 dogs, 10 were castrated males and 19 were spayed females. Most patients (18/29) were diagnosed with urothelial carcinoma based on *BRAF* mutation testing and urine cytology. Cytology alone was used to diagnose 9/29 cases, cytology with a bladder tumor antigen test 1/29 case, and cystoscopic biopsy 1/29 case. Tumor location was distributed, as follows: tumors located in the urinary bladder only (16/29), bladder and urethra (3/29), bladder and prostate (4/29), or urethra only (6/29). One patient had ureteral obstruction at the time of diagnosis due to distal ureter invasion. At the time of initial treatment, suspected distant metastasis was observed in 1 patient. Other patient characteristics are summarized in Table 1.

**Table 1.** Study population details.

No.	Breed	Age (years)	Sex	Body weight (kg)	Tumor site	Response^*^	Number of TACE sessions	Additional treatment	Additional interventions	Survival time (days)	Outcome	Pre-treatment volume (cm^3^)	Post-treatment volume (cm^3^)			Tumor volume reduction (%)	
1	Poodle	7.58	MN	6.7	B, P	PR	4	NSAIDs Chemotherapy		472	Tumor-related death (lung metastasis)	37.5823	14.6742			60.95	
2	Maltese	13.83	MN	7.4	B	PR	2	NSAIDs		606	Non-tumor-related death	3.4032	0.8862			73.96	
3	Poodle	11.67	FS	5.2	U	PR	4	NSAIDs	Urethral stent	503	Tumor-related death (hydronephrosis)	3.8267	0.0953			97.51	
4	Chihuahua	10.75	MN	4.96	B, P	PR	4	NSAIDs Chemotherapy PRT	Ureteral stent (right)	696	Tumor-related death (lung, peritoneal cavity metastases)	1.2584	0.3239			74.26	
5	Maltese	9.83	MN	5.42	B	PR	2	NSAIDs		211	Non-tumor-related death	0.6285	0.0619			90.15	
6	Maltese	10.67	MN	3.96	B	PR	1	NSAIDs PRT	Ureteral stent (right)	865	Alive	1.181	0.0674			94.29	
7	Pomeranian	9.08	FS	2.6	B	PR	2	NSAIDs		569	Non-tumor-related death	0.1545	0.0061985			95.99	
8	Chihuahua	8.83	MN	4.96	B	PR	4	NSAIDs		439	Tumor-related death (hydronephrosis)	1.0704	0.0304			97.16	
9	Bichon Frise	13.42	FS	5.45	U	PR	3	NSAIDs	Cystostomy tube	301	Tumor-related death (chronic pyelonephritis)	0.5287	0.2709			48.76	
10	Mix	7	FS	11.58	B	PR	2	NSAIDs Chemotherapy		742	Alive	1.4114	0.0087817			99.38	
11	Maltese	13.5	MN	3.22	B, P	PR	3	NSAIDs Chemotherapy		338	Tumor-related death (hydronephrosis)	4.7448	1.6576			65.06	
12	Chihuahua	9.92	MN	5.24	B, P	PR	4	NSAIDs Chemotherapy		789	Non-tumor-related death	0.9005	0.04			95.56	
13	Mix	11	FS	4.24	B, U	PR	3	NSAIDs	Urethral stent Ureteral stent (left)	509	Tumor-related death (chronic pyelonephritis)	5.6389	1.2502			77.83	
14	Maltese	9.42	FS	4	B	PR	2	NSAIDs	Ureteral stent (bilateral)	818	Alive	0.5281	0.242			54.18	
15	Maltese	13.58	FS	6.2	B	PR	2	NSAIDs Chemotherapy		563	Non-tumor-related death	3.9767	2.2436			43.58	
16	Poodle	15.42	FS	5.7	B, U	PR	2	NSAIDs	Urethral stent	817	Non-tumor-related death	9.1895	2.1597			76.50	
17	Pomeranian	12.08	FS	3.56	U	PR	4	NSAIDs		677	Non-tumor-related death	1.2674	0.1601			87.37	
18	Pomeranian	5.67	FS	5.1	B	PR	2	NSAIDs Chemotherapy DRT		810	Alive	4.8503	1.0877			77.57	
19	ST	11.58	FS	5.3	U	PR	2	NSAIDs Chemotherapy		582	Alive	1.3219	0.3118			76.41	
20	YT	11	MN	5	B	PR	2	NSAIDs Chemotherapy		207	Tumor-related death (hydronephrosis)	23.8692	5.1527			78.41	
21	Dachshund	7	FS	9.3	B	PR	3	NSAIDs Chemotherapy	Ureteral stent (Left)	561	Tumor-related death (pyonephrosis)	1.8856	1.2236			35.11	
22	Maltese	11.33	FS	4.06	B	PR	2	NSAIDs Chemotherapy		494	Alive	0.6698	0.2703			59.64	
23	C. Spaniel	10.75	FS	13	U	PR	3	NSAIDs	Urethral stent	534	Tumor-related death (bone metastasis)	1.9001	0.2662			85.99	
24	Maltese	12.17	FS	4	U	PR	2	NSAIDs		582	Alive	0.8955	0.196			78.11	
25	Pomeranian	10.17	FS	3.49	B	PR	2	NSAIDs		572	Tumor-related death (lung metastasis)	0.74987	0.416267			44.49	
26	Pomeranian	9.25	FS	4.1	B	PR	4	NSAIDs Chemotherapy		628	Alive	17.0706	5.1437			69.87	
27	Mini-pinscher	7.5	FS	6.1	B	PR	2	NSAIDs Chemotherapy		400	Tumor-related death (urethral obstruction)	5.6276	1.767			68.60	
28	Chihuahua	8.25	MN	4.28	B, U	SD	2	NSAIDs	Ureteral stent (left)	184	Tumor-related death (hydronephrosis of right kidney)	2.608		2.1588			17.22
29	Maltese	12.75	FS	5.03	B	PR	2	NSAIDs Chemotherapy		440	Tumor-related death (hydronephrosis, pyelonephritis)	1.4392		0.8133	43.49		

^*^ Outcome at 1 month after the initial TACE.

### 2.3. Medical records review

Data on the following variables were collected: signalment (age, sex, and breed), body weight, clinical signs at the time of staging, tumor location, history of systemic chemotherapy, cytologic or histopathologic findings, additional diagnostic test results, diagnostic imaging findings, tumor volume measurements pre-treatment and 1 month post-treatment, TACE procedural details, procedure and anesthesia duration, procedural and post-procedural complications, and survival time.

### 2.4. Treatment protocol

All patients were scheduled to undergo at least one TACE procedure. The embolization endpoint was defined as the disappearance of tumor blush on digital subtraction angiography (DSA) while ensuring parent artery patency. This approach was adopted to prevent serious complications, such as ureteral branch ischemia or bladder necrosis, which could occur with excessive embolization.

TACE was performed under general anesthesia, by a single veterinarian. Pre-anesthesia medication included 0.2 mg/kg dexamethasone disodium phosphate (Jeil Dexamethasone, Jeil, Daegu, Korea) and 25 mg/kg cefazolin sodium (Cefazolin, Chongkundang, Seoul, Korea) administered intravenously. For sedation and analgesia, 0.05 mg/kg hydromorphone HCl (Dilid inj., Hana Pharmaceutical Co., Ltd., Seoul, Korea) and 0.2 mg/kg midazolam (Midacum Inj., Myung Moon, Seoul, Korea) were administered intravenously. General anesthesia was induced via intravenous injection of 1% propofol (Provive Inj., Pharmbio Korea, Seoul, Korea), 3–6 mL per dog. Endotracheal intubation was performed, and anesthesia was maintained using isoflurane and oxygen. A crystalloid fluid solution was administered intravenously for intra-operative fluid management. Continuous monitoring included clinical evaluation; inspiratory and expiratory fractions of CO_2_, O_2_, and isoflurane; pulse oximetry; indirect blood pressure measurement; and esophageal or rectal temperature monitoring.

**Figure 1.**
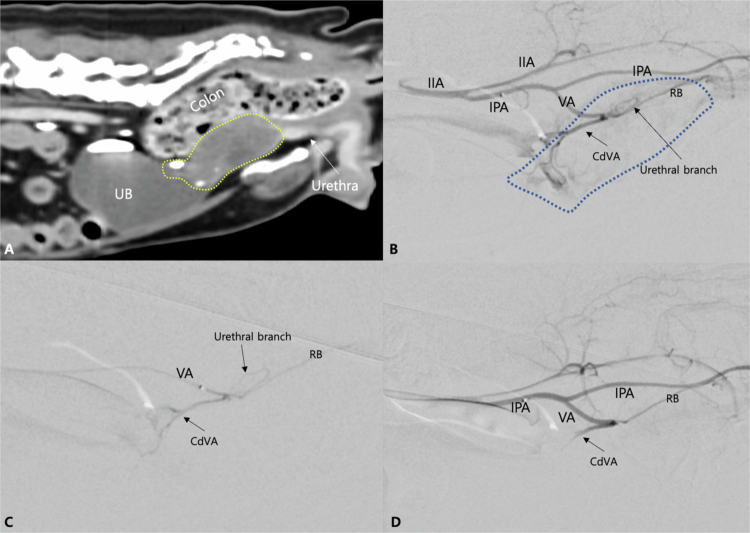
Sagittal reformatting computed tomography (CT) image (A) and intra-operative conventional digital subtraction angiography (cDSA) images (B–D) of a patient diagnosed with urethra and urinary bladder neck urothelial carcinoma (Case No. 16). (A) Post-contrast CT image exhibits markedly thickened proximal urethra with obliteration of the normal urethral mucosal enhancement and extension to the bladder neck (yellow dotted line). (B) Internal iliac artery (IIA) angiography reveals the internal pudendal artery (IPA) and vaginal artery (VA) branching from the IIA. The tumor blush (blue dotted line) is predominantly supplied by the caudal vesical artery (CdVA) and the urethral branch of the VA. (C) During embolic agent infusion, the embolic-contrast mixture is visible within the VA and its branches. (D) Selective angiogram post-embolization, the abnormal tumor blush is no longer observed, and patency of the parent arteries, including the CdVA and rectal branch (RB), is preserved.

All patients were placed in dorsal recumbency, and the carotid regions were clipped and prepared under sterile conditions. A transarterial cut-down approach was performed via the carotid or femoral arteries using a 4 or 5F introducer sheath (Prelude, Merit Medical, South Jordan, USA). For carotid access, a 4F angled angiographic catheter (KMP, Jungsung Medical, Seoul, Korea) was introduced using a 0.035-inch guide wire (Zip Wire, Boston Scientific, Marlborough, USA) through the carotid artery and thoracic aorta, up to the caudal aorta. For femoral access, a 4F J-curved angiographic catheter (MPL, Jungsung Medical, Seoul, Korea) was introduced using a 0.035-inch guide wire through the femoral artery, up to the caudal aorta. The guidewire was withdrawn from the angiographic catheter, and under mobile C-arm fluoroscopy (OEC Elite CFD, GE Healthcare, Chicago, USA) or ceiling-mounted angiography (Artis Q Ceiling, Siemens Healthineers, Erlangen, Germany) guidance, 3–5 mL of a 50% saline/50% contrast mixture (Omnipaque 300^®^, GE Healthcare, Shanghai, China) was injected to visualize the anatomy of the terminal aorta. Superselection of the tumor-feeders was performed using a 0.014-inch microwire (Meister S14, Asahi Intecc, Nagoya, Japan) and 1.5F microcatheter (Veloute ultra, Asahi Intecc, Nagoya, Japan) to access the internal iliac and pudendal arteries (Figure 1). Pre-operative CT (Somatom Scope, Siemens Medical Systems, Forchheim, Germany) or intra-operative cone-beam computed tomography (CBCT) (DynaCT, Siemens Healthineers, Erlangen, Germany) scanning was used to assess the vascular anatomy of the pelvic region. In cases with atypical branching patterns of the internal iliac artery, pre-operative CT or intra-operative CBCT angiographic images were reviewed to guide selective catheterization of the tumor-feeders. In male dogs, based on the tumor location, the caudal vesical and/or prostatic arteries were selectively catheterized. In female dogs, the caudal vesical and/or vaginal arteries were selectively catheterized, depending on the tumoral vascular supply. The microwire-microcatheter combination was subsequently advanced to the terminal arteries, the microwire was withdrawn from the microcatheter, and 1 ml of contrast agent was injected to delineate the terminal arteries.

Prior to embolization, systemic dose of carboplatin (Neoplatin Inj., Boryung Pharma, Seoul, Korea) was administered via the target artery. Subsequently, the tumor-feeders were selectively embolized. For embolization, 100 mg of low-molecular-weight gelatin microparticles (Smart-Gel Mirip 50–125 µm, PL Micromed, Yangsan, Korea) were dissolved in 5 mL of normal saline and mixed with 10 mL of iodinated contrast medium. The mixture was slowly infused into each target artery using gentle manual pressure, until the embolization endpoint. Upon the completion of embolization in all target vessels, the microcatheter, angiographic catheter, and introducer sheath were removed. The carotid or femoral artery was ligated, and the skin incision was closed using routine techniques.

The patients were discharged on the day of the procedure or the day after the procedure. To manage post-procedural discomfort and prevent potential complications such as post-embolization syndrome, the following medications were prescribed for 1 week: 2 mg/kg maropitant, once daily (Cerenia, Zoetis, Kalamazoo, USA), 10 mg/kg gabapentin, twice daily (Neurontin, Viatris, Seoul, Korea), and 12.5 mg/kg amoxicillin-clavulanic acid, twice daily (Amocla, Kuhnil, Seoul, Korea).

### 2.5. Assessment of tumor response

All patients underwent follow-up CT scanning approximately 28 days post-TACE to assess therapeutic response. Pre- and post-treatment CT images were reconstructed using a soft tissue window setting (window width and level: 400 and 40 Hounsfield units, respectively) with a slice thickness of 2 mm. Tumor volume was measured using post-contrast axial CT images using a commercial DICOM viewer (OsiriX MD version 14.1, Pixmeo SARL, Bernex, Switzerland). Measurements were obtained by manual contouring of the tumor margins on each slice, and the volume was calculated automatically by the software. The procedural response rate was classified according to the response evaluation criteria in solid tumors (RECIST) guidelines (Therasse et al. 2000). Complete response (CR) was defined as the disappearance of all target lesions, partial remission (PR) if the longest diameter of tumor on CT transverse images decreased by ≥ 30%, progressive disease (PD) as an increased in tumor diameter of ≥ 20%, and SD as neither meeting the criteria for PR nor PD. The remission rate was calculated as the percentage of patients with CR or PR. The CBR was defined as the percentage of patients with CR, PR, and SD (CR + PR + SD).

### 2.6. Assessment of clinical signs and follow-up

Follow-up was conducted by telephone with the patients’ owners on day 2 and by in-person clinical visits on day 14 post-embolization and 1 month post-procedure to evaluate treatment outcomes and any complications. On day 14, a CBC was performed to monitor for potential bone marrow suppression due to intra-arterial carboplatin administration. In cases where the owners were unable to return to the hospital, the CBC was assessed at a local primary veterinary clinic, and results were shared. Neutropenia, if detected, was classified as grade 1 (1500–2799 cells/μL), grade 2 (1000–1499 cells/μL), grade 3 (500–999 cells/μL), or grade 4 (<500 cells/μL) (Veterinary Co-operative Oncology 2004). Any changes in clinical signs (hematuria, dysuria, appetite, and activity level) and adverse effects were systematically recorded during each follow-up.

### 2.7. Complications

Intra- and post-procedural complications were recorded for all patients. Post-discharge complications within two weeks after the procedure were assessed via telephonic follow-up with the owners. Complications were classified follows: minor (adverse events manageable on an outpatient basis) and major (adverse events requiring hospitalization, urgent intervention, or potentially threatening to the patient’s life or function).

### 2.8. Statistical analysis

Survival time was defined as the duration from the date of the first TACE to the date of death or the last follow-up. Descriptive statistics were expressed as median and range. Individual tumor volume reduction was calculated for each dog as follows: {(Pre-treatment volume − Post-treatment volume)/Pre-treatment volume} × 100 (%). The Kaplan–Meier survival curves were constructed to visualize survival distributions. In addition, MSTs were calculated for each subgroup based on subsequent treatment modalities following initial therapy and tumor locations, and log-rank tests were used to assess between-group statistical significance. For comparisons of tumor volume pre- and post-embolization, the Wilcoxon matched-pairs signed-rank test was applied. Changes in binary clinical signs were summarized using paired transition tables and analyzed using the exact McNemar test. The percentage and 95% confidence interval (CI) for change in tumor volume were calculated. All statistical analyses were two-sided, and a *p* value < 0.05 was considered statistically significant. Data were analyzed using statistical software (SPSS Version 29.0.2.0, IBM Corp., Armonk, USA).

## 3. Results

### 3.1. Treatment protocol

**Figure 2.**
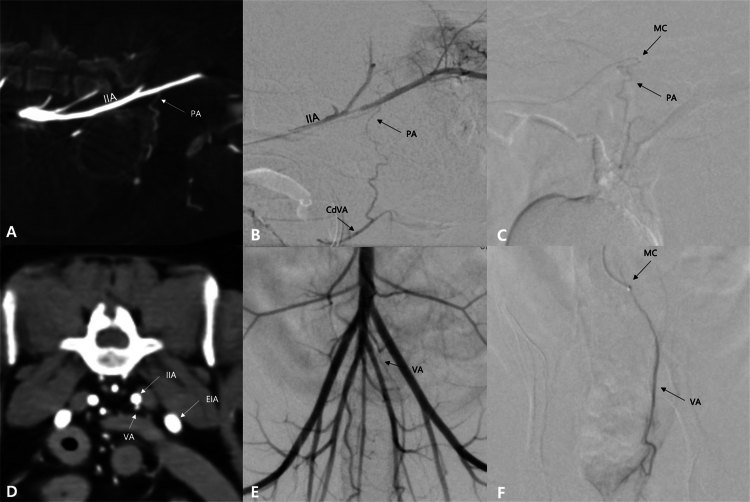
Computed tomography (CT) and fluoroscopy images of 2 patients (Case No. 28: A–C; No. 7: D–F) demonstrating variations in internal iliac artery (IIA) anatomy. (A–C) The left prostatic artery (PA) originates directly from the IIA, as visualized in the maximal intensity projection CT image (A) and selective angiogram of the IIA (B). A super-selective angiogram of the aberrant PA (C) reveals a tortuous PA and tumor blush enhancement. (D–F) The left vaginal artery (VA) originates from the umbilical artery, as visualized in the transverse CT image (D) and selective angiogram of the distal aorta (E). A super-selective angiogram of the aberrant VA (F) reveals a mildly dilated vessel diameter and tumor enhancement. EIA: external iliac artery, MC: microcatheter tip.

Initial TACE was technically successful in all patients, indicating that vascular access was achieved, and the major tumor-feeders included the caudal vesical and prostatic or vaginal arteries. Variations in the internal iliac artery anatomy were identified using DSA images in 3 patients (Figure 2): vaginal or prostatic artery originating from the umbilical artery (*n* = 2, unilateral) and that originating directly from the internal iliac artery (*n* = 1, unilateral). The median duration for TACE was 59 min (range, 45–110 min), and the median duration of anesthesia was 82 min (range, 56–146 min). The median total volume of the embolic slurry infused per patient was 0.7 mL (range, 0.35–2.1 mL).

### 3.2. Assessment of tumor response

**Figure 3.**
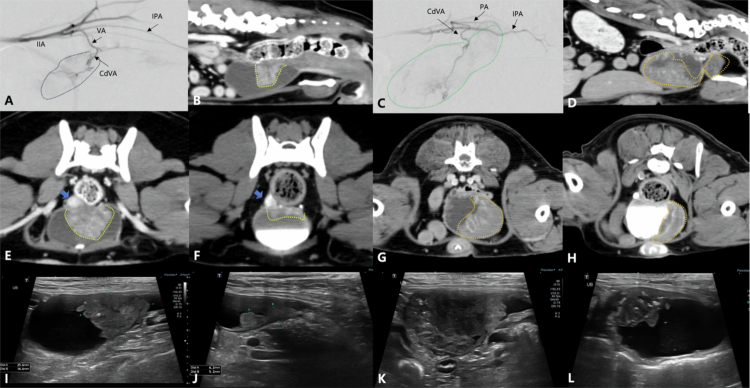
Representative imaging findings in 2 patients diagnosed with urothelial carcinoma. (A, B, E, F, I, J) Images from Case No. 18 with urothelial carcinoma involving the urinary bladder. (A) Intra-operative conventional digital subtraction angiography (cDSA) image reveals the tumor blush (blue dotted line) supplied by the caudal vesical artery (CdVA). (B, E) Pre-operative computed tomography (CT) images reveal a single heterogenous enhancing mass (yellow dotted line) originating from the caudoventral urinary bladder wall with right ureteral invasion (blue arrow). (I) Pre-operative sagittal ultrasonography image reveals an iso- to hyperechoic mass with irregular margin. (F, J) Follow-up CT (F) and sagittal ultrasonography (J) images obtained 1 month post-embolization reveal a marked reduction in tumor size. (C, D, G, H, K, L) Images from Case No. 20 with urothelial carcinoma involving the urinary bladder and prostate. (C) Intra-operative cDSA image reveals the tumor blush (green dotted line) supplied by the CdVA and prostatic artery (PA). (D, G) Pre-operative CT images reveal a diffuse irregularly marginated mass involving the urinary bladder and prostate with mild contrast enhancement (orange dotted line). (K) Pre-operative sagittal ultrasonography image reveals a hypoechoic mass in the urinary bladder with irregular margin. (H, L) Follow-up CT (H) and sagittal ultrasonography image (L) obtained 1 month post-embolization reveal tumor size reduction with focal mineralization. IIA: internal iliac artery, IPA: internal pudendal artery.

**Figure 4.**
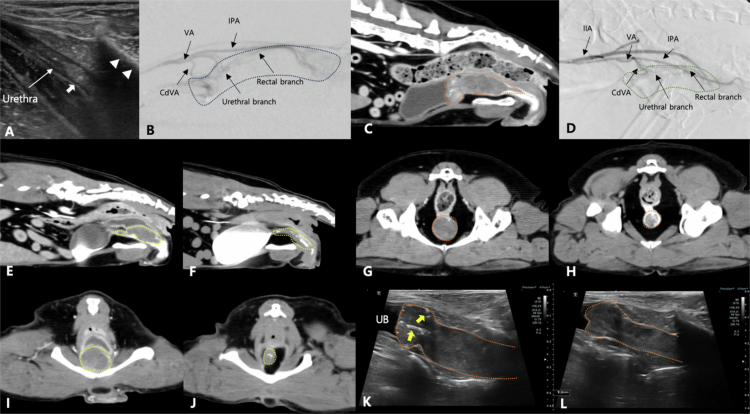
Representative imaging findings in 2 patients diagnosed with urothelial carcinoma. (A, B, E, F, I, J) Images from Case No. 3 with urothelial carcinoma involving the urethra. (A) Ultrasonography images reveal thickening of the proximal urethra with hyperechoic foci (white arrow). The middle and distal parts of the urethra are not visible due to acoustic shadowing from the pubis (white arrow heads). (B) Intra-operative cDSA image reveals tumor blush (blue dotted line), which is supplied by the caudal vesical artery (CdVA), and rectal and urethra branches from the vaginal artery (VA). (C, I) Pre-operative computed tomography (CT) images demonstrate a hypoattenuating urethral mass (yellow dotted line), especially in the mid-to-distal region, with enhancement at the margin after contrast administration. (F, J) Follow-up CT images obtained 1 month post-embolization reveal tumor size reduction with focal mineralization. (C, D, G, H, K, L) Images from Case No. 16 with urothelial carcinoma involving the urinary bladder and urethra. (C and G) Pre-operative CT images demonstrate a large hypoattenuating mass (orange dotted line) in the urinary bladder neck and urethra. The mass extends to the middle of the urethra. (D) Intra-operative cDSA image reveals tumor blush (green dotted line), which is supplied by the CdVA, and rectal and urethral branches from the VA. (K) Pre-operative sagittal ultrasonography image reveals a hypoechoic mass in the urinary bladder and urethra, with mineralization at the bladder neck (yellow arrows). (H, L) Follow-up CT (H) and sagittal ultrasonography images (L), obtained 1 month post-embolization, reveal a reduction in tumor size. The ultrasonography images (K, L) have the same depth of field. IIA: internal iliac artery, IPA: internal pudendal artery.

**Figure 5.**
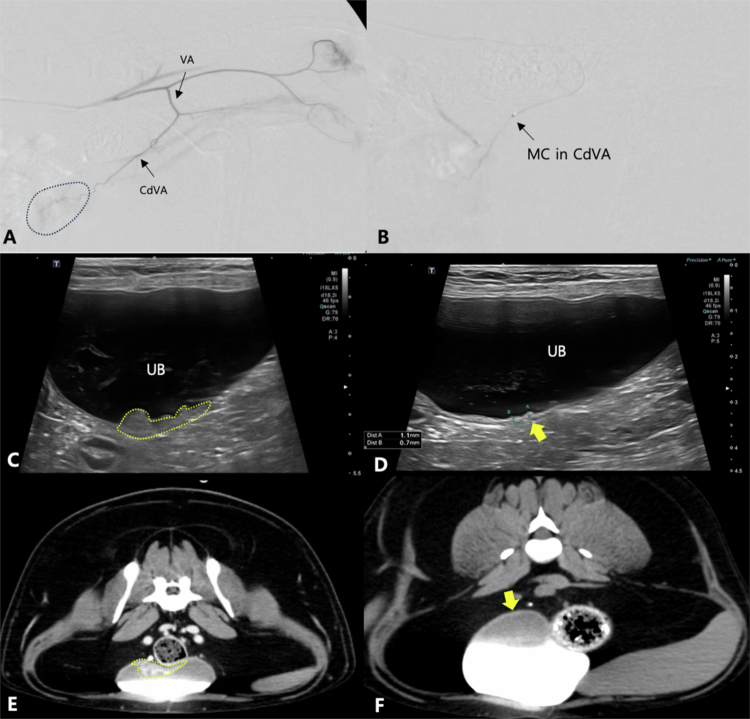
Representative imaging findings of the patient (Case No. 10) with the most favorable therapeutic response, diagnosed with urothelial carcinoma involving the dorsal wall of the urinary bladder. (A, B) Intra-operative conventional digital subtraction angiography images reveal tumor blush (blue dotted line) supplied by the caudal vesical artery (CdVA). The distal segment of the CdVA is selectively catheterized using a microcatheter (MC), and the embolic agent administered. (C, E) Pre-operative ultrasonography (C) and computed tomography (CT) (E) images demonstrate an irregularly marginated mass (yellow dotted line) in the dorsal bladder wall. (D, J) Follow-up ultrasonography and CT images obtained 1 month post-embolization reveal a marked reduction in tumor size. Most of the mass has regressed, with only focal wall thickening remaining (yellow arrow). The patient remains alive 742 days post-treatment with complete resolution of clinical signs.

**Figure 6.**
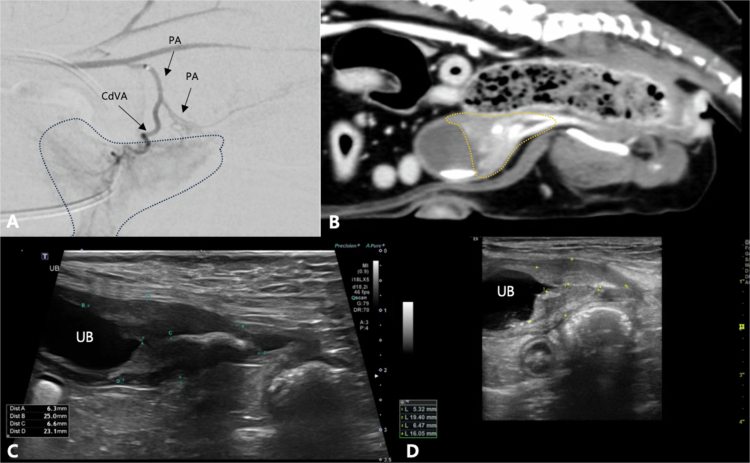
Representative images of a patient with urothelial carcinoma involving the urinary bladder neck minimal treatment response (stable disease) (Case No. 28). (A) Intra-operative cDSA reveals a distinct tumor blush (dotted blue line) supplied by the caudal vesical artery (CdVA). The CdVA appears dilated and tortuous compared with the prostatic artery (PA) branch. (B) Pre-operative CT image reveals an irregularly marginated mass with moderate contrast enhancement, occupying most of the bladder neck lumen. (C, D) Ultrasonography images obtained pre-operatively (C) and 1 month post-embolization (D) demonstrate a mild reduction in tumor size in the sagittal plane. However, the tumor occupies most of the bladder neck. The patient survived for 184 days and died due to hydronephrosis secondary to ureteral invasion of the tumor.

All patients demonstrated a reduction in tumor size on CT scans performed 4 weeks post-embolization (Figures 3 and 4). In paired within-dog comparisons, tumor volumes were significantly reduced following embolization (Wilcoxon matched-pairs signed-rank test, *p* < 0.001). The pre- and post-embolization median tumor volumes were 1.4392 cm^3^ (range: 0.1545–37.5823 cm^3^) and 0.3239 cm^3^ (range: 0.0062–14.6742 cm^3^), respectively. The median tumor volume loss was 76.41% (95% CI, 65.06–78.41%). PR was observed in 28/29 patients, and 1 patient exhibited SD with a 22.4% reduction in the longest tumor diameter (Figures 5 and 6). The median reduction in the longest tumor diameter was 50.93% (95% CI, 46.44–57.73%). The patients classified as SD exhibited a tumor volume reduction of 17.22%. At 1-month post-procedure, the remission rate and CBR were 96.55% and 100%, respectively.

### 3.3. Assessment of clinical signs and follow-up

Clinical signs reported pre-embolization included hematuria, pollakiuria, stranguria, nocturia, anorexia, lethargy, and urinary incontinence (Table 2). By day 28 post-embolization, most clinical signs had improved, with a statistically significant improvement in pollakiuria, hematuria and stranguria. Additionally, nocturia, anorexia, and lethargy, although observed in only a few patients, resolved post-procedure. However, urinary incontinence persisted in 1 patient with a bladder and urethral tumor, with no improvement observed post-embolization. CBC performed 2 weeks post-procedure revealed neutropenia in 4 patients: grade 1 in 1 patient, grade 2 in 2 patients, and grade 4 in 1 patient. The pre-embolization and 2 weeks post-embolization hematological parameters are summarized in Table 3.

**Table 2.** Paired transition analysis of clinical signs in 29 dogs before and 28 days after TACE.

Clinical sign	Persisted	Improved	Worsened	Absent both	*P* value
Pollakiuria	9	12	0	8	<0.001
Hematuria	0	19	1	9	<0.0001
Stranguria	1	14	0	14	<0.001
Nocturia	0	3	0	26	0.25
Anorexia	0	2	0	27	0.50
Lethargy	0	1	0	28	1.00
Incontinence	1	0	0	28	NA

TACE, transarterial chemoembolization; NA: not applicable; Persisted: Pre +/Post +; Improved: Pre +/Post-; Worsened: Pre-/Post+, Absent both: Pre-/Post.

**Table 3.** Hematological parameters of the patients before and 2 weeks after embolization.

Parameters (ref. range)	Pre-embolization median (range)	2 weeks post-embolization median (range)
RBC (6.54–12.2 M/μl)	7.01 (5.16–9.16)	6.71 (4.75–8.88)
HCT (30.3–52.3%)	45.2 (33.0–58.5)	42.6 (28.3–59.5)
WBC (2.87–17.02 k/μl)	9.83 (4.09–23.03)	8.93 (1.15–17.59)
NPH (1.15–10.29 k/μl)	6.76 (1.9–8.36)	6.1 (0.23–15.4)
PLT (151–600 k/μl)	409 (5.83–913)	342 (121–660)

Data are presented as median and range.

RBC, red blood cell count; HCT, hematocrit; WBC, white blood cell count; NPH, neutrophil count; PLT, platelet count.

### 3.4. Complications

TACE was safely performed without any intra-operative complications. No major adverse events such as bladder ischemia, perforation, or procedure-related mortality were observed. Mild post-procedural complications included transient pain (*n* = 9/29), anorexia (*n* = 8/29), and lethargy (*n* = 2/29), all of which resolved within 3 days post-procedure. One patient exhibited hematochezia between days 3 and 5, presumably associated with embolization of the rectal branch, which improved with supportive treatment. Another patient experienced a single seizure episode of < 1 min on day 2 post-procedure, which was not recurrent, with no further neurological abnormalities being observed. No gastrointestinal adverse effects such as vomiting or diarrhea, associated with intra-arterial chemotherapy infusion, were observed.

### 3.5. Long-term outcomes

During the follow-up period, 28/29 (96.6%) patients underwent additional TACE after the initial procedure: 1 session (3.4%), 2 sessions (55.2%), 3 sessions (17.2%), and 4 sessions (24.1%). Additional TACE was performed when tumor regrowth or clinical deterioration was observed. In the absence of follow-up CT scans after the additional TACE, ultrasonographic comparisons demonstrated tumor size reduction in all cases.

Regarding adjunct therapies, 14 (48.3%) patients received only oral NSAIDs post-TACE. Twelve (41.3%) patients were administered systemic chemotherapy, including carboplatin (*n* = 2), vinblastine (*n* = 4), mitoxantrone (*n* = 1), or a combination protocol (*n* = 5). RT was administered in 3 (10.3%) patients: NSAIDs with palliative radiation therapy (PRT) (*n* = 1), NSAIDs combined with systemic chemotherapy and PRT (*n* = 1), and NSAIDs, systemic chemotherapy, and definitive radiation therapy (DRT) (*n* = 1).

**Figure 7.**
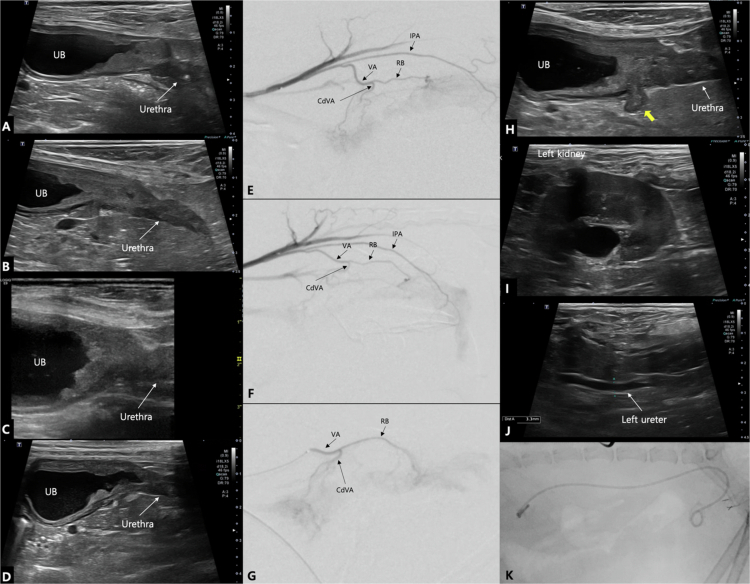
Ultrasonography and conventional digital subtraction angiography (cDSA) images from the initial treatment to 13 months post-treatment (Case No. 13). (A, E) Ultrasonography and right internal iliac angiogram images obtained at the time of the first transarterial chemoembolization (TACE). (B, F) Images obtained at the second TACE, 2 months later. (C, G) Ultrasonography and right vaginal artery angiogram images obtained at the third TACE, 7 months after the first TACE. In all cDSA images, the tumor-feeders and recanalization of the parent arteries are clearly visualized. (D) Follow-up ultrasonography obtained 1 month after the final embolization reveals a reduction in tumor size, in the sagittal plane. (H) At 13 months after the initial TACE, the ultrasonography image reveals progression of the tumor with invasion into the left ureter (yellow arrow), resulting in hydronephrosis and hydroureter (I, J). A percutaneous ureteral stent placed to resolve the obstruction (K). The patient survived for 509 days and died due to pyelonephritis and sepsis secondary to ascending urinary tract infection.

Ten patients required additional interventional procedures during the follow-up period. Six patients developed ureteral obstruction and hydronephrosis due to tumor invasion at the ureterovesical junction and underwent percutaneous ureteral stenting (Figure 7). Among these, 1 patient subsequently developed urethral obstruction, necessitating additional urethral stenting. In 4 others, urethral obstruction occurred independently: 3 underwent urethral stent placement and 1 received a cystostomy tube.

**Figure 8.**
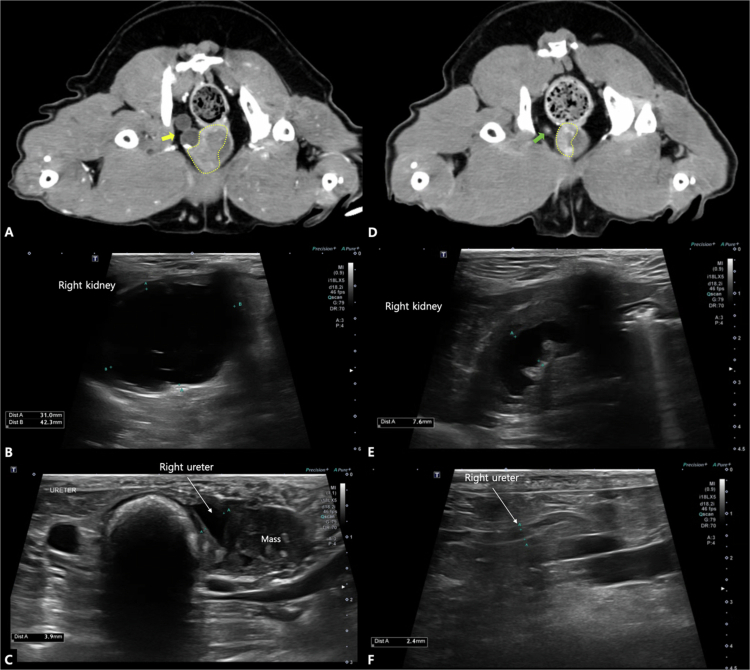
Pre-procedural and 1-month after the initial transarterial chemoembolization (TACE) computed tomography (CT) and ultrasonography images (Case No. 27). (A, D) Transverse CT images. Pre-procedural image (A) reveals a bladder trigone mass (yellow dotted line) with right ureteral invasion and dilation (yellow arrow). One month post-TACE (D), the tumor size has reduced, with a decrease in the diameter of the right ureter (green arrow). (B, C, E, F) Pre-procedural ultrasonography images (B, C) reveal hydronephrosis and hydroureter, which exhibit improvement on follow-up ultrasonography 1 month post-procedure (E, F).

At the time of initial treatment, 3 patients (Case No. 6, 27, and 28) had hydronephrosis due to ureteral invasion by the tumor. Among them, in 1 patient (Case No. 27) hydronephrosis consequent to relieved ureteral obstruction following tumor size reduction (Figure 8). However, in the remaining 2 patients (Case No. 6 and 28), despite the reduction in tumor size post-TACE, hydronephrosis did not improve.

Of the 29 patients monitored for > 1 year, 21 died during the study period. Among these, 7 deaths were unrelated to tumor progression: chronic kidney disease (*n* = 3), diabetic ketoacidosis and hyperosmolar hyperglycemic syndrome (*n* = 1), left atrial tear secondary to myxomatous mitral valve disease (*n* = 1), acute congestive pulmonary edema secondary to myxomatous mitral valve disease (*n* = 1), and systemic inflammatory response syndrome due to acute pancreatitis (*n* = 1). The remaining 14 deaths were attributable to tumor progression: renal failure due to ureteral obstruction and hydronephrosis (*n* = 6), systemic inflammation due to pyelonephritis or pyonephrosis (*n* = 2), urethral obstruction (*n* = 1) and metastases (lungs, *n* = 3; bones, *n* = 2). Necropsy was not performed in these cases because owner consent could not be obtained.

**Figure 9.**
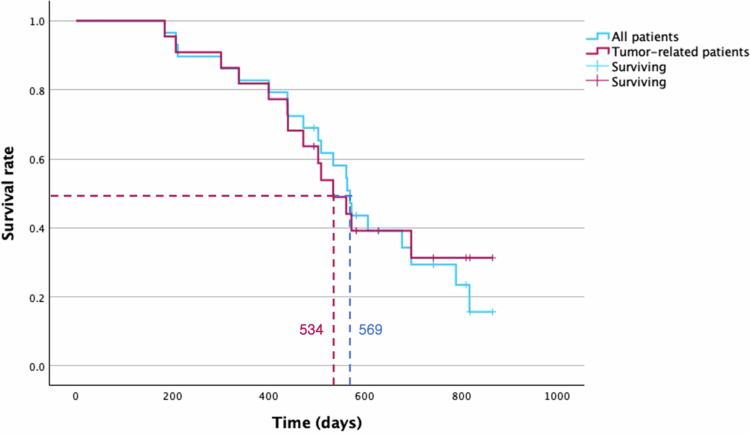
Kaplan–Meier survival curves of all treated patients (blue) and patients excluding those with non-tumor-related deaths (red). The median survival time (MST) for all treated patients was 569 days, whereas, non-tumor-related deaths were excluded, the MST decreased to 534 days for deaths due to tumor progression.

**Figure 10.**
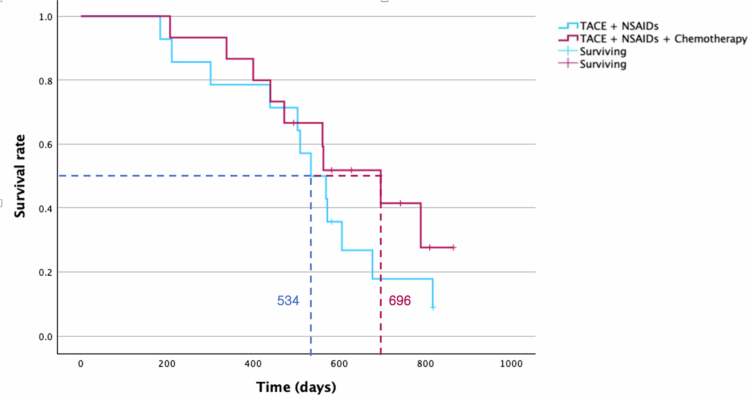
Kaplan–Meier survival curves comparing patients treated with transarterial chemoembolization (TACE) and nonsteroidal anti-inflammatory drugs (NSAIDs) (blue) and those treated with TACE and NSAIDs combined with systemic chemotherapy (red). Patients treated with NSAIDs and chemotherapy post-TACE had a longer median survival time (MST) (696 days) than those treated with NSAIDs (534 days). The difference is not statistically significant (*p* = 0.307).

The MST for all treated patients was 569 days (95% CI: 520.70–617.30) after the initial TACE (Figure 9). When non-tumor-related deaths were excluded, the MST decreased to 534 days (95% CI: 450.08–617.92) for deaths due to tumor progression. Among subgroups, patients treated with NSAIDs and chemotherapy post-TACE had a longer MST (696 days, 95% CI: 423.99–644.00) than those treated with NSAIDs medication (523 days, 95% CI: 486.56–905.44) (*p* = 0.307) (Figure 10).

Patients with tumors localized to a single anatomic site, such as the bladder or urethra alone, exhibited a longer MST (569 days, 95% CI: 552.87–585.13) than those with tumors involving multiple sites (509 days, 95% CI: 414.05–603.95), including the bladder and urethra or bladder and prostate (*p* = 0.393). Among the 22 patients with single-location tumors, 8 (36.4%) were alive at the time of analysis, compared with 0/7 (0%) with multi-location involvement. The surviving dogs had a median follow-up time of 685 days (range, 494–865 days).

## 4. Discussion

This study investigated using TACE with QS-GMP to manage lower urinary tract tumors in dogs, aiming to alleviate clinical signs, reduce tumor size, and assess procedural safety. The procedure was well-tolerated, with no major complications such as bladder ischemia, perforation, or uroabdomen. Technical success was achieved in 100% of patients, including in the small-breed dogs (2.6 kg). Clinical signs, particularly stranguria and hematuria, improved in most patients. Follow-up CT imaging performed 4 weeks after embolization demonstrated tumor size reduction in all cases, with a CBR of 100% (PR, *n* = 28/29; SD, *n* = 1/29).

TACE with quick-soluble embolic agents is a minimally invasive procedure that occludes microvasculature via a catheter at the target vessels. The concept was first introduced by Okuno et al. in 2013 for palliation of musculoskeletal pain, using imipenem/cilastatin (IPM/CS) as the embolic agent. IPM/CS particles, with an average size of approximately 130 µm, induce transient embolization and are reportedly safe for temporary occlusion without causing permanent ischemic damage (Okuno et al. 2013). Similar to chronic inflammation-induced neovascularization, tumor-associated neovessels are vulnerable to transient embolic occlusion (Koucheki et al. 2021). Tumor endothelial cells differ structurally from those of normal vasculature as follows: (1) the endothelium forms a disorganized monolayer with protrusions (trabeculae) into the vascular lumen; (2) intercellular gaps and a defective basement membrane, which facilitate blood leakage into the interstitium; and (3) abnormal vascular sprouts that arise from the tumor endothelial cells (Dudley 2012). These aberrant neovessels, owing to their architectural instability and deficiency, are more susceptible to irreversible collapse post-embolization, even when temporary embolic agents are used. Thus, embolization can lead to persistent luminal obliteration and a marked reduction in tumor hypervascularity, resulting in tumor regression.

In this study, the clinical outcomes demonstrated significant therapeutic efficacy and an excellent safety profile. Furthermore, since recanalization of the parent arteries occurred, without permanent vessel damage, repeat embolization procedures were feasible. In this study, 28/29 patients underwent additional embolization, with 7 (24.1%) receiving up to 4 treatments. The repeat procedures allowed for sustained palliation of clinical signs and tumor control, maintaining quality of life.

In human medicine, most embolization procedures related to the lower urinary tract have focused on bladder vessel embolization for intractable hemorrhage and prostatic arterial embolization for benign prostatic hyperplasia and hemorrhage (Delgal et al. 2010; Abt et al. 2013; Kim et al. 2021). The most common complications are minor, and include post-embolization syndrome and gluteal pain (Chen et al. 2021). However, the use of permanent embolic agents includes a risk of non-target embolization in the internal iliac arterial territory, with possible severe adverse effects (e.g. bladder ischemia or necrosis) (Delgal et al. 2010; Tarkhanov et al. 2018). In veterinary medicine, embolization with permanent embolic agents such as polyvinyl alcohol microspheres or drug-eluting beads has been reported for prostatic tumors (Culp et al. 2021; Pellerin et al. 2021). No major or minor complications, including post-embolization syndrome, was reported. Owing to the risk of serious complications consequent to embolic agent leakage into the bladder, embolization has been limited to the prostatic arteries. In contrast, our study demonstrated that QS-GMP enables safe embolization in bladder and urethral malignancies, dissolving approximately 12 h post-infusion and enabling recanalization within 48 h (Kim et al. 2022). This temporary embolic effect is sufficient to disrupt aberrant neovessels while facilitating early recanalization of normal vasculature, thereby effectively minimizing ischemic injury to the bladder wall.

In veterinary medicine, several embolic agents, including gelatin sponge particle, microspheres, and polyvinyl alcohol, have been used in TAE and TACE (Marioni-Henry et al. 2007; Delfs et al. 2009; Oishi et al. 2019; Culp et al. 2021; Rogatko et al. 2021; Kimata et al. 2022; Nakasumi et al. 2022). However, consensus regarding the optimal embolic agent in veterinary medicine remains lacking. Conversely, with their demonstrated safety and efficacy (temporary vascular occlusion with a reduced risk of ischemia), novel quick-soluble embolic agents, such as QS-GMP and resorbable microsphere, are increasingly used in human medicine (Min et al. 2023; Okuno 2023; van Zadelhoff et al. 2025). However, quick-soluble embolic agents are yet to be applied in veterinary medicine. This study is first to report their use in veterinary practice. The results revealed that temporary vessel occlusion using QS-GMP was associated with a low rate of complications and effective tumor response. Moreover, the recanalization of parent arteries permitted repeat embolization. Although this study focused solely on urinary tract tumors, the favorable outcomes suggest that these agents may be useful in other embolization procedures in veterinary medicine, particularly in cases where non-target embolization is a concern.

Treatments for canine urothelial carcinoma include surgery, radiotherapy, oral NSAIDs, chemotherapy, and local intravesical therapy. Surgical intervention may help alleviate clinical signs and has been associated with longer MST than those with medical treatments alone (Marvel et al. 2017; Bennett et al. 2018). However, canine lower urinary tract tumors commonly involve critical regions such as the bladder trigone or ureters, requiring extensive procedures like trigonal excision, subtotal or total cystectomy, and grafting for bladder reconstruction (Hautmann et al. 2006; Zhang et al. 2006; Saulnier-Troff et al. 2008; Boston and Singh 2014; Fulkerson and Knapp 2015; Saeki et al. 2015). Despite their description in the literature, the high morbidity risks and costs associated with such surgical techniques preclude their widespread application in clinical practice.

Systemic chemotherapy and oral NSAIDs are commonly used to treat urogenital carcinomas. A study investigating piroxicam monotherapy reported a CBR of 77.6% (PR, 18.4%; SD, 59.2%) and an MST of 244 days (Knapp et al. 1994). When systemic chemotherapy was combined with NSAIDs, the MST was 291–329 days (Fulkerson and Knapp 2015). Other chemotherapeutic agents include mitoxantrone, carboplatin, vinblastine, and cisplatin (Mutsaers et al. 2003; Vail et al. 2007). In a clinical trial, piroxicam + mitoxantrone combination therapy achieved a CBR of 78% (PR, 8%; SD, 69%) and an MST of 247.5 days, and a piroxicam + carboplatin combination therapy achieved a CBR of 67% (PR, 13%; SD, 54%) and an MST of 263 days (Allstadt et al. 2015). Oral tyrosine kinase inhibitors have been investigated for the treatment of canine urothelial carcinoma. These include toceranib, a multi-targeted receptor tyrosine kinase inhibitor, and lapatinib, a dual kinase inhibitor that selectively targets both epidermal growth factor receptor and human epidermal growth factor receptor 2 (HER2) (Gustafson and Biller 2019; Maeda et al. 2022). Lapatinib has demonstrated favorable outcomes particularly in HER2-overexpressing tumors. In a clinical trial, lapatinib + piroxicam combination therapy achieved a CBR of 66% (CR, 2%; PR, 52%; SD, 12%) and an MST of 435 days, suggesting superior efficacy over conventional medical treatments (Maeda et al. 2022). In the present study, the CBR of 100% indicates superior outcomes, a notable, 28 patients achieving PR (96%).

Despite requiring general anesthesia, TACE with QS-GMP remains minimally invasive and directly contributes to tumor volume reduction. A study on intra-arterial chemotherapy revealed a significantly higher tumor response than conventional intravenous chemotherapy, with a CBR of 100% (PR, 36%; SD, 64%) and minimal adverse effects, although the long-term outcomes were not assessed (Culp et al. 2015). Similarly, in the present study, combining intra-arterial chemotherapy with embolization possibly enhanced therapeutic efficacy. In the present study, the tumor response appeared more favorable than that reported in the intra-arterial chemotherapy only study, with a CBR of 100% (PR, 96%; SD, 4%). This enhanced response may reflect the combined therapeutic effect of carboplatin and tumor embolization. Additionally, a follow-up CBC performed 2 weeks post-procedure revealed neutropenia in 4/28 patients. However, no associated clinical signs such as diarrhea, vomiting, or alopecia were observed.

External beam radiotherapy has been incorporated into definitive treatment protocols to improve locoregional tumor control. Advanced modalities such as intensity-modulated radiotherapy and volumetric modulated arc therapy, with image guidance, which offer superior conformality and minimized radiation exposure to organs at risk, enhance treatment efficacy and tolerability. A CBR of 90% in dogs with urogenital carcinomas, with an MST of 654 days, has been reported with RT (Nolan et al. 2012). Another study involving 51 dogs with urogenital carcinoma treated with RT, with or without additional medical treatment, reported an overall MST of 510 days (Clerc-Renaud et al. 2021). Radiotherapy-related adverse effects include skin damage, pain, pollakiuria, urinary incontinence, cystitis, stranguria, and hydronephrosis (Ahrens et al. 2024). In the present study, the overall MST was 569 days, demonstrating a long-term prognosis comparable to that of radiotherapy, with the advantage of minimal treatment-related complications. In addition, most patients exhibited improvement in clinical signs within 7 days, contributing to the maintenance of quality of life. Three patients received adjunctive radiotherapy based on the owner’s preference: two received PRT (5 fractions, once weekly for 5 weeks) and one received DRT (15 fractions). One PRT-treated patient developed hydronephrosis post-RT and subsequently underwent percutaneous ureteral stent placement. The DRT-treated patient remains alive at 810 days. One PRT-treated patient died on day 696, and the other remains alive at day 865; combining radiotherapy with systemic chemotherapy and TACE may yield extended survival.

According to previous preclinical animal and human clinical studies, embolization-induced tumor necrosis may potentially lead to hematogenous dissemination from the primary tumor (Bonfil et al. 1988; Fang et al. 2013). In addition, a higher incidence of pulmonary metastasis following TACE has been reported in patients with hepatocellular carcinoma (Liou et al. 1995). That study suggested that pulmonary metastasis occur earlier in patients treated with TACE compared with untreated patients. Several mechanisms have been proposed to explain this phenomenon. Tumor embolization can induce tumor hypoxia, which increases the expression of hypoxia-inducible factor-1α within tumor tissues (Chen et al. 2012). This factor may promote a more aggressive tumor phenotype and facilitate metastatic progression. Another proposed mechanism is an increase in circulating tumor cells after embolization, which may enhance the likelihood of distant metastatic seeding (Fang et al. 2013). In the present study, only one dog had detectable distant metastasis at the time of the embolization procedure. This patient survived for 301 days, and although the pulmonary nodules increased in size and number during follow-up, they were not considered the direct cause of death. Distant metastasis was identified as the primary cause of death in five of the 29 dogs; however, these events occurred relatively long after the final embolization procedure, making it difficult to determine whether embolization directly contributed to metastatic progression. Future studies comparing embolization-treated patients with non-treated control groups are needed to further clarify this potential risk in canine lower urinary tract tumor patients. In addition, combining embolization with adjunctive systemic therapies, such as chemotherapy, may help reduce the risk of metastatic progression.

In this study, 3 patients presented with hydronephrosis secondary to ureteral obstruction before TACE. Although hydronephrosis persisted in 2 patients, the obstruction resolved in the third patient post-embolization. Canine urothelial neoplasia is frequently complicated by local tumor invasion leading to urethral and/or ureteral obstruction (Fulkerson and Knapp 2015). Once ureteral obstruction and associated renal impairment are identified, therapeutic options are limited and typically include percutaneous ureteral stenting, subcutaneous ureteral bypass via laparotomy, radical surgery, hemodialysis, or nephrostomy (Berent et al. 2011; 2012; Wormser et al. 2015). Among these, nephrostomy and hemodialysis offer only short-term management, i.e. temporary decompression, rather than resolution for ureteral obstruction. Subcutaneous ureteral bypass or ureteral stenting via laparotomy are less favored because of their invasiveness and the potential for tumor seeding due to bladder incision or ureteral bypass devices (Merickel et al. 2021). Conventionally, percutaneous ureteral stenting remains the most widely accepted and appropriate approach (Berent et al. 2011; Gibson et al. 2021). However, as demonstrated in this study, TACE with QS-GMP successfully relieved ureteral obstruction in 1 patient with remission. Repeat TACE may be considered in the event of recurrence. If therapeutic response is inadequate, percutaneous ureteral stenting remains a viable alternative. In the 2 patients where embolization did not resolve hydronephrosis, percutaneous ureteral stents were placed to relieve the obstruction.

In this study, 3 patients exhibited anatomical variations of the internal iliac artery. The prostatic or vaginal artery most commonly originates from the internal pudendal artery (91.4%), and anatomical variations include direct origin from the internal iliac (5.17%) or umbilical (3.45%) arteries (Avedillo et al. 2016). Similarly, in the present study, in 1 patient, a unilateral prostatic artery originated directly from the internal iliac artery, whereas in 2 patients, a unilateral prostatic or vaginal artery originated from the umbilical artery. In all 3 patients, successful selective catheterization using 1.5F microcatheter enabled embolization.

## 5. Limitation

This study had several limitations. First, tumor response evaluation was limited to the short-term period, as long-term follow-up was challenging owing to variability in additional treatment protocols selected by the owners and clinicians. Second, serial CT imaging for long-term monitoring was not feasible because general anesthesia was required, which posed procedural risks. Although ultrasonography was used in follow-up tumor size monitoring, it only offered 2-dimensional measurements and was prone to operator-dependent variability, potentially reducing the objectivity of tumor response assessment. Third, the potential confounding effect of concomitant medications, particularly NSAIDs, should be considered. To minimize treatment-related interference, dogs that had received systemic chemotherapy within one month prior to enrollment were excluded. However, NSAIDs were permitted up to the time of embolization because they represent standard-of-care baseline therapy for most dogs with urothelial carcinoma. Although NSAIDs may have anti-angiogenic effects and may improve clinical signs, discontinuation prior to enrollment was not considered clinically appropriate due to the risk of worsening clinical signs. Lastly, the study lacked reference control groups such as systemic chemotherapy, NSAIDs alone, or radiotherapy, limiting direct comparison of therapeutic efficacy across treatment modalities. In addition, because embolization and intra-arterial carboplatin infusion were performed concurrently, it was not possible to clearly determine whether the observed tumor reduction was attributable to embolization alone, carboplatin alone, or the combined effect of both treatments. Although this study demonstrated favorable outcomes with TACE, future studies should validate the long-term benefits and comparative efficacy of this approach by incorporating standardized treatment groups and objective imaging protocols.

## 6. Conclusion

This prospective study demonstrated the feasibility and safety of TACE for canine lower urinary tract carcinomas, demonstrating excellent short-term clinical outcomes without serious complications. Moreover, repeat TACE was feasible in cases of tumor regrowth or recurrence of clinical signs, allowing management of clinical signs. TACE achieved a longer MST, comparable to that of RT. Thus, TACE may improve quality of life dogs with lower urinary tract carcinomas via effective tumor control with minimal adverse effects.

## Data Availability

The data that support the findings of this study are available from the corresponding author, D.C., upon reasonable request.
